# Klotho, the Greek goddess controlling the fate of skeletal muscle satellite cells

**DOI:** 10.1113/EP091515

**Published:** 2023-10-26

**Authors:** Mark C. Turner

**Affiliations:** ^1^ Centre for Health and Life Sciences, Institute for Health and Wellbeing Coventry University Coventry UK

**Keywords:** differentiation, exercise, mechanical loading, myogenesis, satellite cells, wnt signalling

1

In humans, skeletal muscle enables the ability to perform a wide variety of tasks for daily living that range from walking to communicating and breathing. However, like other tissues and organs, ageing or disease can lead to a decline in skeletal muscle health and, consequently, impair function. Therefore, understanding what causes a decline in skeletal muscle health and how to mitigate it in order to maintain a high quality of life with increasing age is important, considering the association with morbidity and mortality (McLeod et al., 2016). Regular and sustained exercise provides a countermeasure to age‐related declines in skeletal muscle mass, strength, metabolism and function, which begin to occur in the third decade of life. As a result, there has been a significant drive by scientists and clinicians to understand how exercise can be used to enhance skeletal muscle health in ageing (Philp & Coen, 2023).

Since its discovery in the late 1990s, Klotho has been identified as an ageing suppressor that prolongs longevity. Expressed in several tissues, including skeletal muscle (Olauson et al., 2017), Klotho increases the affinity binding of fibroblast growth factors (FGFs), such as FGF19, FGF21 and FGF23, to their respective receptors (Kuro‐o, 2019). In consequence, Klotho has been considered a biomarker and potential therapy for age‐related disorders, such as chronic kidney disease, diabetes *mellitus* and cancer (Kuro‐o, 2019). In the context of skeletal muscle physiology, Klotho has been found to be an antagonist of the Wnt signalling pathway, which is important in the formation and development of skeletal muscle. Arguably, it is for this reason that Klotho has caught the attention of skeletal muscle physiologists seeking to understand its role in skeletal muscle stem cell fate, regeneration and growth (Ahrens et al., 2018; Welc et al., 2020).

In this issue of *Experimental Physiology*, Ochi et al. (2023) sought to establish the role of Klotho in skeletal muscle myogenesis in vitro using C2C12 murine myoblast cells and in vivo using a Klotho transgenic mouse model exposed to mechanical loading and high‐intensity interval training respectively. In the in vitro experiments, myoblast cells exposed to mechanical loading combined with exogenous Klotho increased the mRNA expression of paired‐box transcription factor 7 (*Pax7*) by >70%. However, the presence of Klotho attenuated the mechanically induced expression of Myogenin (*Myog*), a crucial myogenic regulatory factor involved in the terminal differentiation of myoblast cells and overall muscle development (Dumont et al., 2015). Further analysis showed that the addition of exogenous Klotho in the medium of mechanically stimulated myoblasts suppressed the mRNA expression of Wnt genes (*Wnt10a* and *Wnt9a*), which are implicated in the switching of cells from proliferation to terminal differentiation. Subsequent in vivo experiments in a Klotho transgenic mouse model undergoing high‐intensity interval training showed similar synergistic responses, with increases in the expression of *Pax7* and myoblast differentiation 1 (*Myod*) in a synergistic fashion with an attenuated *Myog* expression, unlike the wild‐type mice, which showed high‐intensity interval training‐induced increases in *Myog*. Supporting the in vitro evidence, Klotho transgenic mice showed attenuated co‐expression of Pax7 and β‐catenin, providing further evidence of the effects of Klotho on canonical Wnt signalling and myogenesis. The in vitro and in vivo findings provide evidence that Klotho can put the brakes on the fate of mechanical or exercise‐induced terminal differentiation of skeletal muscle myoblasts.

How does the research by Ochi and colleagues fit within the broader context? Skeletal muscle myogenesis is a tightly regulated process of proliferation and differentiation of skeletal muscle stem cells, or satellite cells (SCs), into myoblasts and, finally, multinucleated muscle fibres. A pluripotent population of cells, SCs reside under the basal lamina and have the ability, upon external stimulation (e.g., exercise), to commit to the myogenic lineage in order to support the maintenance and repair of existing muscle fibres or to generate new ones (Dumont et al., 2015). Ageing has been shown to have an effect on the number and function of SCs; however, like many of the cellular and molecular responses to exercise, the SC pool has the ability to expand its population of myogenic cells which have the potential to terminally differentiate and contribute to skeletal muscle growth and repair in young and old individuals (Snijders et al., 2015). Therefore, targeting or enhancing circulating Klotho, in order to expand the SC pool with proliferating myogenic cells has its benefits if the SC pool is diminished or if there is attenuated functional capacity, particularly in ageing muscle where the expansion of the SC pool in response to exercise is delayed or attenuated in comparison to younger individuals (Snijders et al., 2015). This raises several potential questions about how the synergistic effects of Klotho and exercise can be put to best use to expand the SC pool of myogenic cells that will, ultimately, have the capacity and ability to differentiate and contribute to maintaining muscle mass as it begins to decline with age (McLeod et al., 2016).

Future research will seek to determine whether the large expansion of myoblast cells, which are produced in the presence of Klotho during mechanical loading or exercise, can be used, as well as how Klotho is implicated in human skeletal muscle ageing and its response to exercise (Correa et al., 2022). Certainly, the findings by Ochi et al. (2023) provide insight into how Klotho can affect mechanically induced skeletal muscle myogenesis, and this research will generate further interest within the field with regard to the function of Klotho and its role in exercise‐induced adaptions of healthy and ageing skeletal muscle.

## AUTHOR CONTRIBUTIONS

Sole author.

## CONFLICT OF INTEREST

The author declares no conflicts of interest.

## FUNDING INFORMATION

None.
